# Efficacy and safety of a NiTi CAR 27 compression ring for end-to-end anastomosis compared with conventional staplers: A real-world analysis in Chinese colorectal cancer patients

**DOI:** 10.6061/clinics/2016(05)04

**Published:** 2016-05

**Authors:** Zhenhai Lu, Jianhong Peng, Cong Li, Fulong Wang, Wu Jiang, Wenhua Fan, Junzhong Lin, Xiaojun Wu, Desen Wan, Zhizhong Pan

**Affiliations:** Sun Yat-Sen University Cancer Center; State Key Laboratory of Oncology in South China, Collaborative Innovation Center of Cancer Medicine, Department of Colorectal Surgery, Guangzhou, P.R., China

**Keywords:** NiTi CAR 27, Anastomosis, Colorectal Cancer, Efficacy, Safety

## Abstract

**OBJECTIVES:**

This study aimed to evaluate the safety and efficacy of a new nickel-titanium shape memory alloy compression anastomosis ring, NiTi CAR 27, in constructing an anastomosis for colorectal cancer resection compared with conventional staples.

**METHODS:**

In total, 234 consecutive patients diagnosed with colorectal cancer receiving sigmoidectomy and anterior resection for end-to-end anastomosis from May 2010 to June 2012 were retrospectively analyzed. The postoperative clinical parameters, postoperative complications and 3-year overall survival in 77 patients using a NiTi CAR 27 compression ring (CAR group) and 157 patients with conventional circular staplers (STA group) were compared.

**RESULTS:**

There were no statistically significant differences between the patients in the two groups in terms of general demographics and tumor features. A clinically apparent anastomotic leak occurred in 2 patients (2.6%) in the CAR group and in 5 patients (3.2%) in the STA group (*p*=0.804). These eight patients received a temporary diverting ileostomy. One patient (1.3%) in the CAR group was diagnosed with anastomotic stricture through an electronic colonoscopy after 3 months postoperatively. The incidence of postoperative intestinal obstruction was comparable between the two groups (*p*=0.192). With a median follow-up duration of 39.6 months, the 3-year overall survival rate was 83.1% in the CAR group and 89.0% in the STA group (*p*=0.152).

**CONCLUSIONS:**

NiTi CAR 27 is safe and effective for colorectal end-to-end anastomosis. Its use is equivalent to that of the conventional circular staplers. This study suggests that NiTi CAR 27 may be a beneficial alternative in colorectal anastomosis in Chinese colorectal cancer patients.

## INTRODUCTION

In China, colorectal cancer (CRC) has become one of the most common malignancies, ranking fifth in males and fourth in females 1. Although surgical resection is the most effective method for curing CRC, postoperative complications such as anastomotic leakage reportedly lead to poor oncologic outcomes such as local recurrence in CRC patients 2,3. Currently, a stapling device is widely used for providing well-established anastomosis, especially for a low anterior resection, as this device can place the purse-string suture on the distal rectum, which is impossible in the hand-sewn procedure 4-6. Nevertheless, anastomotic complications, such as anastomotic bleeding, strictures and leakages, still occur postoperatively 7-9. Additionally, the localized inflammatory response accounts for the foreign bodies that cross the mucosal barriers and thus evoke potential anastomosis-related morbidity 10,11.

Recently, new compression devices incorporating nickel-titanium shape memory alloys (NiTi CAR 27) have been applied for end-to-end colorectal anastomosis. Unlike the traditional staplers, NiTi CAR 27 provides sutureless colorectal anastomosis by using compression, which may thus avoid anastomotic complications resulting from inflammatory processes due to the suture equipment 11. The safety of NiTi CAR 27 has been demonstrated in numerous animal studies 12,13. Early clinical research also suggested that NiTi CAR 27 might create a more secure colorectal anastomosis and serve as an effective alternative for anastomotic procedures 14,15. To date, the clinical outcome after the application of NiTi CAR™27 on CRC Chinese patients remains unclear. The aim of this single-center study is to evaluate the safety and efficacy of a NiTi CAR 27 device in an end-to-end anastomosis after sigmoidectomy and anterior resection by comparing the complication rates and the long-term postoperative survival data with stapled anastomosis.

## METHODS

### Patients

In this retrospective study, consecutive patients undergoing sigmoidectomy and anterior resection with NiTi CAR 27 were included from May 2010 to June 2012 at the Cancer Center, Sun Yat-Sen University, Guangzhou, China. The clinical data of CRC in the similar locations resected using stapled anastomosis (STA) were collected during the same period. The inclusion criteria were as follows: age >18 years, Eastern Cooperative Oncology Group (ECOG) performance status ≤2, pathological diagnosis of adenocarcinoma, and rectum tumor located ≥3 cm above the anal verge. The exclusion criteria were as follows: tumor located on the right side of the colon, sigmoidectomy and an anterior resection performed previously, uncontrolled severe cardiovascular and respiratory system disease, pregnancy, and a history of other active malignancies (except for basal cell carcinoma of the skin) during the previous 3 years. NiTi CAR 27 was applied for the end-to-end anastomosis by a team of surgeons who have been using stapling anastomotic devices for more than 10 years in our department. The pathological stage of the tumor in the resected specimen was classified according to the tumor-node-metastasis (TNM) staging system of the American Joint Committee on Cancer/International Union Against Cancer (AJCC/UICC) 16. Prior to the surgical procedure, informed consent was obtained from all of the patients. They were well informed of the method of anastomotic construction by NiTi CAR 27. Study approval was obtained from independent ethics committees at the Cancer Center of Sun Yat-Sen University. The study was undertaken in accordance with the ethical standards of the World Medical Association Declaration of Helsinki.

### Evaluation of clinical parameters

The clinical data on the patient demographics, tumor features, perioperative parameters, and postoperative complications were recorded with an electronic medical record system. The perioperative parameters included the length of the operation time, the perioperative blood loss during the hospital stay, and the time to the first flatus. Clinical anastomotic leakage was diagnosed based on symptoms such as fever, abdominal pain and tenderness. The presence of fecal discharge from the pelvic drain or fecal bubbles surrounding the anastomosis observed in computed tomography scans was also recognized as anastomotic leakage. Anastomotic stricture was well defined as the diameter of the lumen that was smaller than a standard 12-mm diameter colonoscopy. Anastomotic hemorrhage was noted when there was an excess anal blood loss of 50 ml postoperatively.

### Description and procedure of using NiTi CAR 27

**Figure 1:**
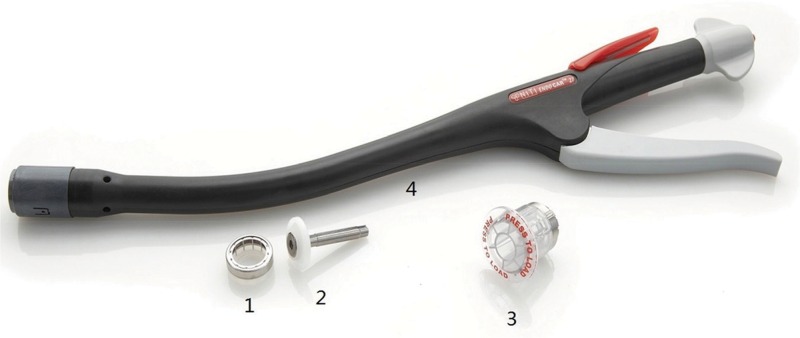
The structure of the NiTi compression anastomosis ring (NiTi CAR 27). It consists of 1. A nitinol mental compression ring, 2. an anvil, 3. a ring loader, and 4. a firing device.

NiTi CAR 27 is composed of a main body and detachable compression elements (Figure 1). The compression elements include a plastic anvil ring and a metal ring incorporating a temperature-dependent shape memory nitinol leaf. The leaf springs maintain a continuous pressure at the anastomosis, independent of the thickness of the tissue. When the tissue around the circular edges heals, the compression element falls off of the compressed tissue within the following 8-10 days as the metal ring is expelled from the body by bowel movement 17. The NiTi CAR 27 was manipulated according to the manufacturer’s instructions. Before the procedure, the nitinol metal ring is saturated in ice water for approximately 30 seconds. After the anvil of the NiTi CAR 27 is secured, the bowel is returned to the peritoneal cavity. Then, the firing device is pushed from the anus to the blind end of the corresponding colorectum. Subsequently, the knob is rotated to the left until the pricker of the firing device reaches a proper position and connects with the anvil. The knob is rotated to the right as a click sound is heard. As a result, end-to-end anastomosis is constructed by pushing forward the firing handle and withdrawing the firing device from the intestinal canal. Finally, the completion of the anastomosis is confirmed by an air-leak test. If the leakage test is positive, a diverting ileostomy is performed.

### Postoperative follow-up

After discharge from the hospital, all patients were followed up in the first month after the operation and every 3 months for the first 2 years postoperatively. Then, an every 6-month follow-up was conducted until 5 years after the surgical operation. The follow-up data were obtained by mail, telephone correspondence and outpatient department visits. The routine follow-up visits consisted of a physical examination, routine blood tests, chest radiography, abdominal ultrasonography (US), contrast-enhanced computed tomography (CT) scans and a pelvic nuclear magnetic resonance (MRI). An electronic colonoscopy was suggested for all patients at the third month postoperatively.

### Statistical analyses

The continuous variables are presented as the mean ± standard deviations and were compared using a Mann–Whitney U-test or Fisher's exact test. Categorical results were compared using a chi-square test. The Kaplan–Meier method was applied to determine the overall survival (OS). Differences in survival outcomes between the two groups were compared with the log-rank test. All of the tests were two-tailed, in which a *p* value <0.05 was considered statistically significant. The data were analyzed using Statistical Package for the Social Sciences (SPSS) 17.0 for Windows (SPSS Inc. Chicago, IL, USA).

## RESULTS

### Clinical characteristics

A total of 234 patients received an anterior resection or a sigmoidectomy for colorectal cancer. The clinical pathological characteristics of the patients from the group with anastomosis using NiTi CAR 27 (CAR group, N=77) and the group with the stapling device (STA group, N=157) were compared (Table 1). There was no significant difference in terms of preoperative clinical demographics and tumor features, including age, sex, clinical stage, body mass index (BMI), tumor sites and comorbidities with the exception of severe anemia. Perioperative parameters, including length of operation time, perioperative blood loss, hospital stay and time to first flatus, were also comparative between the two groups (Table 2).

### Postoperative complications

**Figure 2:**
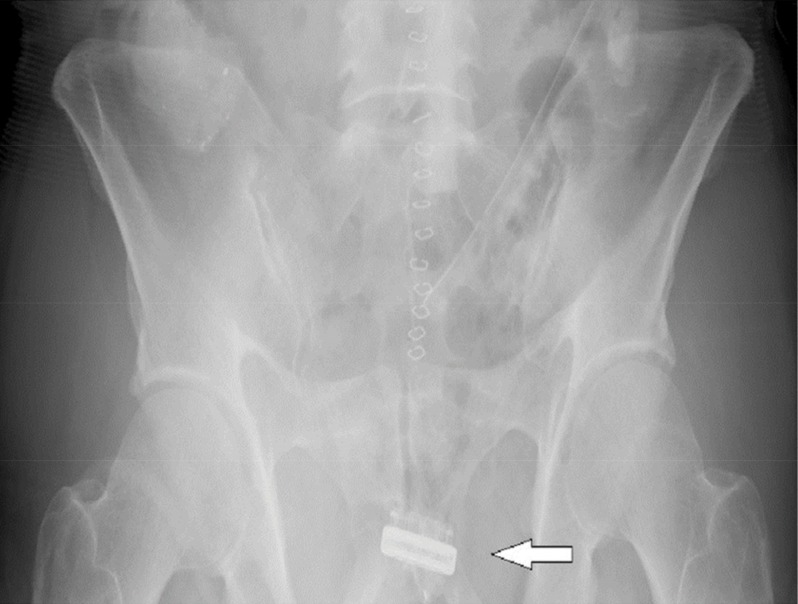
Abdominal radiographs showing the preliminary healing of anastomotic leakage after 5 days post-operation with the ring (arrow) retained in the anastomosis before the ileostomy takedown.

**Figure 3:**
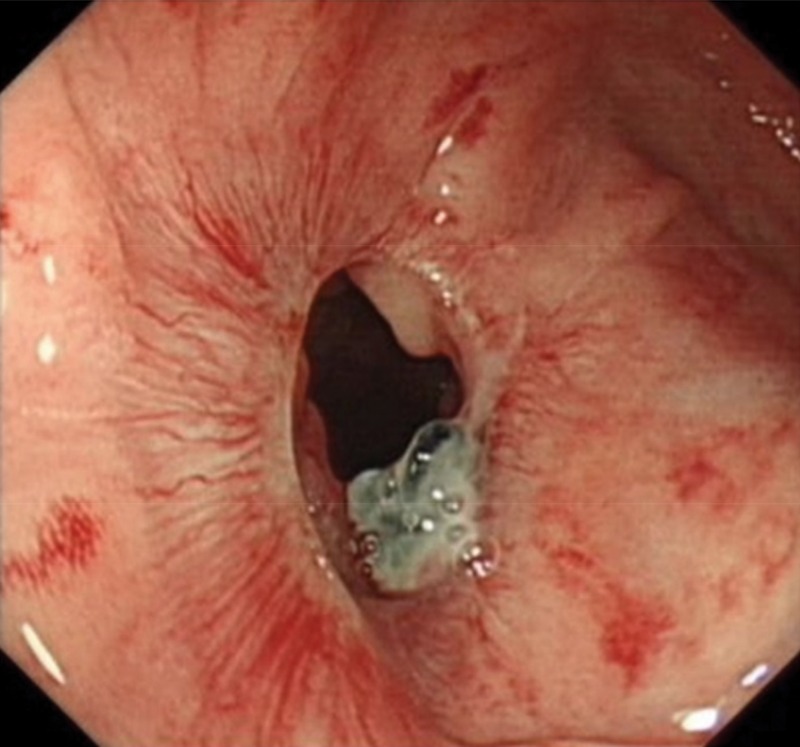
One patient experienced anastomosis stricture after 3 months post-operation as determined by an electronic colonoscopy.

**Figure 4:**
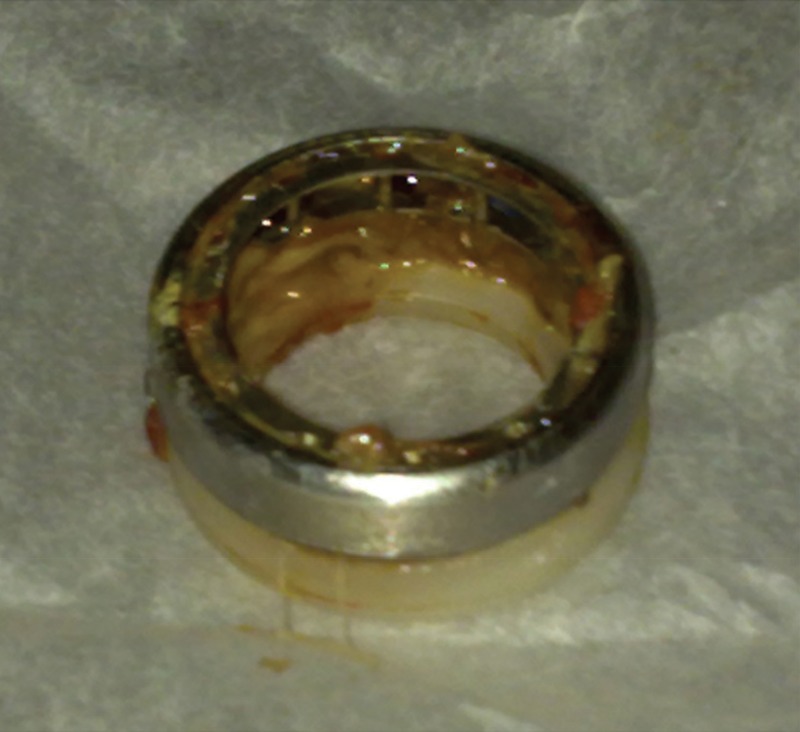
The evacuated NiTi compression anastomosis ring (NiTi CAR 27).

**Figure 5:**
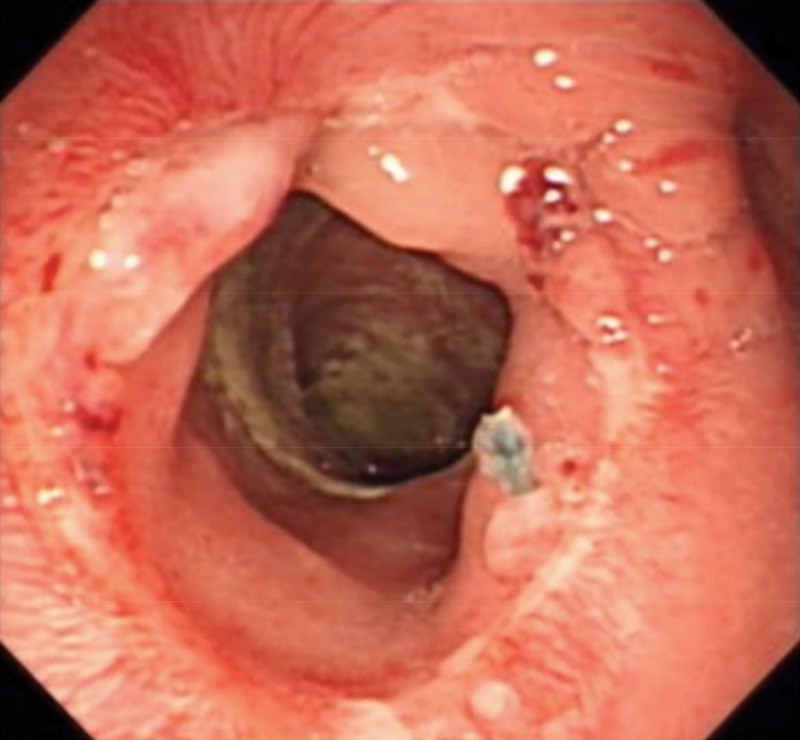
The electronic colonoscopy view of the smooth anastomosis after 3 months postoperatively.

The incidence of postoperative complications, such as anastomotic leakage, anastomotic stricture and intestinal obstruction, did not show significant differences between the two groups (Table 3). Detailed information on the six patients who experienced postoperative complications in the CAR group is summarized in Table 4. A clinically apparent anastomotic leakage occurred in 2 patients (2.6%) (Figure 2). A 46-year-old female undergoing a low anterior resection for rectal cancer (5 cm from the anus) experienced anastomotic leakage on the 4^th^ postoperative day. The other case of leakage was observed in a 61-year-old male who received an anterior resection for rectal cancer (10 cm from the anus) within 5 days after the surgery. A temporary diverting ileostomy was performed for these two patients. Consequently, the symptoms of both patients were relieved after surgery. Anastomotic stricture was diagnosed by an electronic colonoscopy after 3 months in a 65-year-old male (1.3%) receiving an anterior resection (Figure 3). No treatments were required due to the absence of obvious symptoms. Three patients (3.9%) experienced intestinal obstruction at 6-11 days postoperatively. A 74-year-old man recovered after conservative treatment, whereas the condition of a 20-year-old woman receiving the same treatment worsened because of organ failure as a result of tumor progression. Another 68-year-old male with postoperative obstruction was successfully treated with a reoperation. The NiTi CAR 27 rings were spontaneously evacuated from the stool within 10 to 20 days (median 14 days) after the operation (Figure 4). The anastomosis was smooth and without metallic foreign material after 3 months when observed using electronic colonoscopy (Figure 5). No anastomotic hemorrhage or perioperative death occurred in this study.

### Long-term outcome

**Figure 6:**
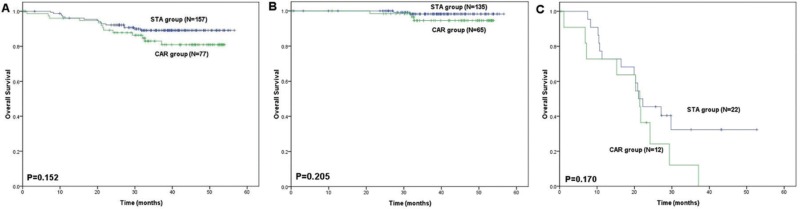
The 3-year overall survival (OS) was 83.1% (CAR group) and 89.0% (STA group), *p*=0.152(A). In the stage 0-III patients, the 3-year OS was 94.5% (CAR group) and 98.2% (STA group), *p*=0.205; (B) in the stage IV patients, the 3-year OS was 12.1% (CAR group) and 32.3% (STA group), *p*=0.170 (C).

With median follow-up duration was 39.6 months. The 3-year overall survival rates in the CAR group and the STA group were 83.1% and 89.0%, respectively (*p*=0.152, Figure 6A). For the stage I-III patients, the 3-year overall survival rates in the CAR group and the STA group were 94.5% and 98.2%, respectively (*p*=0.205, Figure 6B). For the stage IV patients, the 3-year overall survival rates in the CAR group and the STA group were 12.1% and 32.3%, respectively (*p*=0.170, Figure 6C).

## DISCUSSION

Nitinol, an advanced metal alloy of nickel and titanium, exhibits “shape memory,” which is the ability to return to its original shape after being deformed according to the temperature 18. NiTi CAR 27, a new device manufactured with this metal alloy, has several advantages over conventional staplers for colorectal anastomosis. First, the nitinol leaf springs adapt to variations in tissue thickness and accommodate the compressed tissue with a constant force around the full circumference of the anastomosis 11. Without a permanent suture remaining, the use of this device reduces the local long-term inflammatory process of anastomosis. Moreover, the scarring area of the anastomosis is minimal and appears smooth during the healing process 19-21. Although the US Food and Drug Administration (FDA) approved NiTi CAR™ 27 for use in intestinal anastomoses in August 2006, clinical experiments using NiTi CAR 27 in colorectal anastomosis are scarce in China. To evaluate the safety and efficacy of using this device for colorectal cancer surgery, we conducted the present study on Chinese patients.

Thus far, most clinical studies on the application of NiTi CAR 27 for colorectal anastomosis have been conducted from a single center, including only elective cases from several countries. The collected information of the postoperative complications from partial clinical trials involving nitinol compression devices for colorectal anastomosis between 2011 and 2014 are shown inTable 4 21-23,17,24,14,25,15. Lee et al. performed a left-sided colon resection with an anastomosis in 79 patients by using NiTi CAR 27. The phase II study showed that only 1 of 79 (1.3%) patients experienced anastomotic leakage after 6 days postoperatively without any clinical symptoms of anastomotic stricture 22. Another study examining the short-term clinical outcome of a phase II prospective study in 23 patients also demonstrated that NiTi CAR 27 is a promising, safe and effective alternative for the creation of left-side colorectal anastomosis with a low risk of morbidities, including 1 case of abscess (4.3%), 1 case of anastomotic leakage (4.3%) and 2 cases of anastomotic strictures (8.7%) 17. In addition, the largest data analysis of 1,180 patients who underwent rectal resection with the NiTi ColonRing for an end-to-end anastomosis from 178 centers in 16 countries revealed a promising clinical result, with only a 3.2% (38 patients) overall anastomotic leakage rate 14. These results suggested that NiTi CAR 27 might be a safe and flexible device for colorectal surgery with a low incidence of postoperative complications.

Compared to stapled anastomosis, the occurrence of general postoperative complications related with anastomosis constructed by the NiTi CAR 27 was not significantly different in our study. Only 2 clinically apparent anastomotic leakages (2.6%) and 1 anastomotic stricture (1.3%) occurred without any anastomotic hemorrhage. As reported in previous clinical studies, anastomotic leakage occurred in 6% to 8% of the individuals with anterior resection by traditional staplers for rectal cancer patients 26-28. Our study indicated that the use of NiTi CAR 27 for colorectal anastomosis resulted in a slightly lower risk of anastomotic leakage when compared to a stapled anastomosis (2.6% *versus* 3.2%, *p*=0.804). A similar result was obtained in another study in lower located rectal cancer (within 6 cm from the anal verge) anterior resection by comparison of stapled and compression anastomosis 15. We observed only 1 patient (1.3%) in the CAR group with anastomotic stricture after anterior resection for rectal cancer at 3 months postoperatively without obvious problems of defecation. Thus far, the cause of anastomotic stricture has not been elucidated. Previous studies have assumed the occurrence of anastomotic stricture was an undetected anastomotic leakage after surgery 25. We considered inadequate blood supply in the anastomosis to also be a potential cause of anastomotic stricture resulting from the persistent pressure by the compression ring. As previous studies have reported, anastomotic hemorrhage was also reduced by using a compression anastomosis ring for colorectal anastomosis 23,25,10. There was no bleeding at the anastomosis site in the CAR group. The compression ring remains in the body to fix the anastomosis for several days and this sustained pressure forced by the compression ring of the NiTi CAR 27 might play a crucial part in preventing bleeding by compressing the small vessels surrounding the anastomosis. It was assumed that it was beneficial to use the NiTi CAR 27 in patients with a high risk of bleeding. To date, the relationship between the compression device and CRC prognosis has not yet been analyzed 29,25. Short-term observations were not sufficient to determine the endpoint of tumor recurrence and progression. Here, we propose the 3-year follow-up survival outcome of the CRC patients who used the NiTi CAR 27. In terms of the comparable 3-year overall survival result between the two groups, we considered that the NiTi CAR 27 obtained a similar long-term survival efficacy for rectal cancer compared with the traditional staplers used for CRC surgery.

There are potential limitations of this study. The first is its retrospective methodology from a single-institution experience. To reduce bias, we only enrolled patients who had the same disease and who underwent the end-to-end anastomosis surgical procedures. Moreover, the limited number of patients in the present study made it difficult to distinguish the slight difference at the level of statistical significance. In addition, the initial treatment for colorectal cancer and the preoperative treatments varied, especially with respect to radiotherapy. These factors, to some extent, influence the occurrence of postoperative complications and long-term survival. However, even with these limitations, the results of our study can be of value for prospective clinical studies in the future.

In conclusion, NiTi CAR 27 is safe and effective for end-to-end anastomosis in colorectal cancer (CRC) surgery with acceptable postoperative complications and shows a similar long-term efficacy in comparison with conventional circular staplers. The use of NiTi CAR 27 is equivalent to that of the conventional circular staplers, suggesting that NiTi CAR 27 might be a beneficial alternative for colorectal anastomosis in Chinese CRC patients.

## AUTHOR CONTRIBUTIONS

Lu Z, Peng J and Li C designed the study, performed the data analysis and wrote the first draft. Pan Z and Wan D were the coordinating investigators for the study and participated in the preparation of manuscript and study protocol. Wang F, Jiang W, Fan W, Lin J and Wu X participated in the data acquisition. All of the authors read and approved the final manuscript.

## Figures and Tables

**Table 1:** Clinical characteristics of the patients in the NiTi CAR 27 and stapler subgroups.

	NiTi CAR 27	Stapler	*p* value
Clinical characteristics	(N=77)	(N=157)	
Gender			0.868
Male	46(59.7%)	92(58.6%)	
Female	31(40.3%)	65(41.4%)	
Median age (year, range)	60(20-77)	59(20-81)	0.885
Median BMI (kg/m^2^)	22.5(14.6 - 29.9)	22.1(16.4 - 29.7)	0.908
Comorbidities			
Hypertension	18(23.4%)	26(16.6%)	0.210
Diabetes mellitus	2(2.6%)	10(6.4%)	0.219
Obesity	20(26.0%)	31(19.7%)	0.278
Severe anemia	2(2.6%)	0(0)	0.043
Intestinal obstruction	1(1.3%)	2(1.3%)	0.987
Preoperative chemoradiotherapy	8(10.4%)	25(15.9%)	0.253
Tumor site			0.938
Sigmoid colon	32(41.6%)	32(40.8%)	
Upper rectum (>5 cm)	41(53.2%)	41(52.9%)	
Lower rectum (≤5 cm)	4(5.2%)	10(6.4%)	
Postoperative pathological stage			0.491
Stage 0	4(5.2%)	7(4.5%)	
Stage I	14(18.2%)	21(13.4%)	
Stage II	27(35.0%)	51(32.5%)	
Stage III	20(26.0%)	56(35.6%)	
Stage IV	12(15.6%)	22(14.0%)	

Abbreviations: BMI: body mass index

**Table 2:** Perioperative data of the patients receiving compression and stapled anastomosis.

Parameter	NiTi CAR 27 (N=77)	Stapler (N=157)	*p* value
Type of operation			
Sigmoidectomy	31(40.3%)	64(40.8%)	0.941
Anterior resection	46(59.7%)	93(59.2%)	
Median operation time (min, range)	140(60-370)	150(60-420)	0.720
Median perioperative blood loss (ml, range)	50(30-1000)	50(20-300)	0.323
Time to the first flatus (day, range)	3(1-7)	3(2-8)	0.149
Median postoperative hospital stay (day, range)	8(6-32)	8(5-29)	0.523

**Table 3:** Comparison of the postoperative complications in two types of anastomotic devices.

Complications	NiTi CAR 27 (N=77)	Stapler (N=157)	*p* value
Anastomotic leakage	2(2.6%)	5(3.2%)	0.804
Anastomotic stricture	1(1.3%)	0(0)	0.152
Intestinal obstruction	3(3.9%)	2(1.3%)	0.192

**Table 4:** Detailed information on the total postoperative complications in the NiTi CAR 27 group.

Gender	Age	Tumor location	Type of operation	Hospital stay (days)	Complication	Complication detected on POD	Measure of diagnose	Intervention	Outcome
female	20	rectum	Anterior resection	27	Intestinal obstruction	11	abdominal X-ray	conservative treatment	deterioration
female	46	rectum	Anterior resection	32	Anastomoticleakage	4	clinical symptoms	Ileostomy	recovery
male	65	rectum	Anterior resection	10	anastomoticstricture	90	electronic colonoscopy	Wait and see	stabilization
male	74	Sigmoid colon	Sigmoidectomy	21	Intestinal obstruction	6	clinical symptoms	conservative treatment	recovery
male	68	Sigmoid colon	Sigmoidectomy	25	Intestinal obstruction	8	abdominal X-ray	Operation	recovery
male	61	rectum	Anterior resection	21	Anastomoticleakage	5	abdominal X-ray	Ileostomy	recovery

Abbreviations: POD: postoperative day

**Table 5:** Summary of the anastomosis-related postoperative complications that occurred in the patients using the nitinol compression devices.

Author(References)	Year	Patients (N)	Median time to first flatus (Day)	Hospital stay (Day)	Anastomotic leakage	Anastomotic hemorrhage	Anastomotic stricture
Lee et al. (22)	2011	79	NR	7 (4-29)	1 (1.3%)	0	0
Buchberg et al. (17)	2011	23	4 (2–31)	5 (3-41)	1 (4.3%)	0	2 (8.7%)
Dauser et al. (23)	2011	62	3 (1-6)	8 (4-183)	0	0	1 (3.3%)
Koo et al. (21)	2012	66	NR	7 (4-20)	1 (1.5%)	0	0
Bernhard et al. (15)	2013	38	NR	11	2 (5.3%)	0	0
Khromov et al. (24)	2013	40	2.4	7.3	2(5%)	0	0
Masoomi et al. (14)	2013	1180	NR	6 (2-21)	38 (3.2%)	NR	NR
Kwag et al. (25)	2014	63	1.7	5.9	1 (1.6%)	0	1 (1.6%)
Present study	2014	79	3 (1-7)	8 (6-32)	2 (2.6%)	0	1 (1.3%)

NR: Not recorded
